# Prolactin Regulates Seasonal Changes in Renal Rheostasis in Djungarian Hamsters

**DOI:** 10.1210/endocr/bqaf117

**Published:** 2025-07-10

**Authors:** Sayantan Sur, Zoe Adam, Timothy A Liddle, Calum Stewart, Irem Denizli, Broderick M B Parks, Leslie S Phillmore, Tyler J Stevenson

**Affiliations:** School of Biodiversity, One Health & Veterinary Medicine, University of Glasgow, Glasgow G61 1QH, UK; School of Biodiversity, One Health & Veterinary Medicine, University of Glasgow, Glasgow G61 1QH, UK; School of Biodiversity, One Health & Veterinary Medicine, University of Glasgow, Glasgow G61 1QH, UK; School of Biodiversity, One Health & Veterinary Medicine, University of Glasgow, Glasgow G61 1QH, UK; School of Biodiversity, One Health & Veterinary Medicine, University of Glasgow, Glasgow G61 1QH, UK; Department of Psychology and Neuroscience, Dalhousie University, Halifax, NS B3H 4R2, Canada; Department of Psychology and Neuroscience, Dalhousie University, Halifax, NS B3H 4R2, Canada; School of Biodiversity, One Health & Veterinary Medicine, University of Glasgow, Glasgow G61 1QH, UK

**Keywords:** kidney, photoperiod, tubule, mammals, transcriptome

## Abstract

Seasonal changes in a photoperiod regulate multiple physiological systems in vertebrates, including metabolism, reproduction, and immune function. Kidney mass and renal physiology are known to vary annually, but the endocrine and molecular mechanisms underlying these changes are poorly defined. Prolactin (PRL), a photosensitive hormone is implicated in seasonal energy rheostasis, yet its role in programmed regulation of renal physiology is unknown. Using Djungarian hamsters (*Phodopus sungorus*), we investigated how photoperiod and PRL regulate seasonal changes in kidney mass, morphology, and transcriptome. Ingestive behavior, kidney histology, and transcriptomic profiles were assessed. We found that long photoperiods and PRL treatment induced renal hypertrophy and convoluted tubule expansion, whereas exposure to short photoperiods resulted in a reduction in all measurements. Transcriptomic analysis revealed photoperiod- and PRL-responsive gene modules related to mitochondrial metabolism, solute transport, and epithelial remodeling. Among these, *Cdh2*, encoding N-cadherin, was downregulated by long photoperiods and PRL, and negatively correlated with convoluted tubule diameter, suggesting a role in epithelial adhesion during tubular expansion. These findings place prolactin as a key hormonal effector for programmed seasonal kidney function and identify *Cdh2* as a target to drive renal physiology.

Seasonally programmed rheostatic changes in energy stability are observed in many animals that inhabit temperate climes (1, 2). Mammals generally exhibit reduced body mass and adipose tissue and engage in torpor during winter dormancy (3). For example, the Djungarian hamster (*Phodopus sungorus*), Golden-mantled ground squirrels (*Callospermophilus lateralis*), and Grizzly bears (*Ursus arctos horribilis*) exhibit energy involution in preparation for hibernation or torpor (4-6). It is now established that a series of discrete neuropeptides in the hypothalamus govern annual changes in energy rheostasis (7). Despite robust and predictable annual cycles in water consumption (5) and kidney mass (8), the mechanisms involved in seasonal changes in renal function remain poorly understood.

The annual change in day length, known as photoperiod, is coded physiologically by nocturnal melatonin secretion by the pineal gland and functions to regulate seasonal physiology in mammals (9, 10). Djungarian hamsters are one of the most well-characterized animal models to investigate the mechanisms that control seasonal physiology (11, 12). In response to short winter photoperiods, hamsters decrease food intake, reduce body mass, and experience adipose and kidney involution (8, 13-17). Water deprivation during winter photoperiods increases the duration of torpor by 1 hour (18). Many neuropeptides involved in energy metabolism and growth such as proopiomelanocortin (*Pomc*) and VGF nerve growth factor inducible (*Vgf*) expression reflect seasonal states; both decrease short photoperiod in Djungarian hamsters (7, 19). In addition, somatostatin (*Sst*) expression in the arcuate nucleus is significantly upregulated in a short photoperiod and implicated in the inhibition of growth (8, 20). However, antidiuretic hormones arginine vasopressin (*Avp*) and oxytocin (*Oxt*) expression in the hypothalamus do not vary across seasonal states in Djungarian hamsters (11, 20) or during torpor (11). In 13-lined ground squirrels, there is an increase in *Avp* expression and the number of genetically active Avp cells in the supraoptic nucleus during torpor (21). These findings indicate that 2 potential mechanisms may govern seasonal water rheostasis: 1 that is species-specific Avp-dependent or an alternative physiological system.

Prolactin (PRL) is an evolutionarily conserved pituitary hormone that regulates several seasonal physiological systems in birds and mammals (22). Increased secretion of PRL in a long summer photoperiod facilitates growth, metabolism, reproduction, energy storage, thermogenesis, and pelage change in mammals (22-25). Prolactin receptors are distributed across multiple tissues, including the kidney (26, 27), and maintain renal homeostasis in vertebrates (22, 28, 29). The role of PRL in water homeostasis was first reported in teleosts, in which hypophysectomized fish retained the ability to adapt to freshwater under PRL treatment (30). In mice, *Prl* expression is localized in the proximal tubules and the Bowman capsule (31). Autoradiography identified PRL binding in the rat renal cortex and medulla, specifically in the epithelial cells of the proximal tubules (32). PRL inhibited the activity of sodium and potassium (Na⁺/K⁺) adenosine 5′-triphosphatase in the proximal segment of the renal tubules of Sprague-Dawley rats (33). Physiological hyperprolactinemia-induced *Prlr* expression in the kidney is proposed to play a key role in regulating calcium homeostasis in female Wistar rats (26). In Djungarian hamsters, acute intraperitoneal injections of PRL significantly increased kidney mass (25). These data indicate that PRL could provide another physiological signal to regulate water rheostasis independent of *Avp* and *Oxt* pathways.

The current study aimed to investigate the molecular architecture of seasonal changes in kidney structure and identify how PRL regulates renal function. Two experiments were conducted to evaluate the impact of photoperiod and PRL on kidney morphology by measuring multiple parameters such as the kidney somatic index, kidney volume, number of renal corpuscles, renal corpuscle area, and perimeter, as well as the convoluted tubule width. Transcriptome sequencing was conducted to identify molecular substrates regulated by photoperiod-induced and PRL-dependent stimuli. As PRL levels naturally peak in summer months (long photoperiod), we hypothesized that the administration of PRL hormone during short photoperiod conditions is sufficient to drive seasonal variation in renal function and identify molecular targets involved in water rheostasis. The data indicate that seasonal variation in PRL drives convoluted tubule diameter changes that are associated with a significant upregulated of *cadherin 2* expression. Overall, the findings show an alternative neuroendocrine pathway links PRL secretion to renal function to regulate annual changes in water rheostasis.

## Material and Methods

### Ethical Approval and Animal Husbandry

The experiments were conducted under the UK Home Office Project License (PP5701950) and approved by the University of Glasgow Animal Welfare and Ethics Review Board. Djungarian hamsters (*Phodopus sungorus*) were group housed in the Veterinary Research Facility at the University of Glasgow under a long-photoperiod (LP) (16 light [L]:8 dark [D]). Food (rodent chow, Harlan Tekland) and water were provided ad libitum.

### Experiments

Two studies were performed to assess the behavioral, morphological, and molecular changes that underlie seasonal changes in water rheostasis. Both male and female hamsters show a photoperiod-induced decrease in circulating prolactin concentrations, indicating that both sexes exhibit the same photoperiodic response. Because female hamsters have variable circulating prolactin concentrations in LP, we opted to use only male hamsters in experiment 1 (34, 35). As both sexes have low circulating prolactin concentrations in short photoperiod, which is an ideal condition to examine the sufficiency of hormone action, we opted to use only females in experiment 2.

#### Experiment 1: photoinduced seasonal changes in water rheostasis

To capture the seasonal change in water intake and renal physiology, we collected hamsters across a simulated seasonal rhythm. Adult male hamsters (N = 54) were housed under LP photoperiod (16L: 8D). A group of hamsters continued under LP conditions (N = 6) as a photoperiod reference group for the duration of the study. The remaining hamsters were exposed to a short photoperiod (SP) (8L: 16D). At 4-week intervals, a subset of hamsters was collected and consisted of animals exposed to SP for 4 weeks (N = 6), 8 weeks (N = 6), 12 weeks (N = 6), 16 weeks (N = 6), 20 weeks (N = 6), 24 weeks (N = 6), 28 weeks (N = 6), and 32 weeks (N = 6) weeks (36). Daily water intake (mL) was measured as a marker of seasonal changes in hydration. On the last week of the photoperiod treatment, water bottles were filled and weighed. Then for 7 consecutive days, the water bottle weight was recorded based on measurements taken by a Toledo SB80001 scale. Daily water intake was calculated by taking the average daily loss in water bottle weight across the 7 days. After photoperiod treatment, hamsters were killed between 2 and 4 hours after lights on by cervical dislocation followed by exsanguination. The kidney was dissected, and dimensions were measured using a vernier caliper. Kidney volume (cm^3^) was calculated using the ellipsoid method, D_1_ × D_2_ × D_3_ × π/6, where D_1_, D_2_, and D_3_ correspond to the length, width, and depth of the kidney (37). Kidneys were then weighed using Sartorius cp64 anatomical balance (0.0001 g) and then stored at −80 °C. The pituitary gland was quickly collected and stored at −80 °C to measure prolactin (*Prl*) mRNA expression.

#### Experiment 2: investigating the role of prolactin in kidney physiology

Next, we sought to examine the sufficiency of circulating prolactin to drive seasonal change in kidney physiology. To reduce the use of animals in research, we took advantage of kidney tissues used from a previous experiment (25). In brief, adult female hamsters (N = 12) were kept in LP photoperiod and then exposed to 8 weeks of SP photoperiod (8L:16D). Animals were then pseudorandomly divided into 2 groups. One group served as a control group and received daily intraperitoneal injections of 100 µL vehicle control (SAL; 50% dimethyl sulfoxide + 50% saline) (N = 6) for 3 consecutive days. The treatment group received PRL (18 µg, NIAMDD-O-PRL) dissolved in the vehicle (N = 6) via intraperitoneal injections of 100 µL for 3 consecutive days. Both groups (SAL and PRL) received injections during the middle of the light phase. On the last day, the hamsters were killed between 2 and 4 hours after lights-on by cervical dislocation followed by exsanguination. Kidney volume and mass were collected as above and then stored at −80 °C.

### Histological Analysis of Kidney

Hematoxylin and eosin staining were performed to identify seasonal and PRL-dependent changes in kidney morphology. Frozen kidneys were cut at a transverse plane using a surgical blade into 2 halves; 1 half was cryosectioned at a 12-μm thickness on a Leica CM1850 cryostat; the other half was stored for molecular analysis. Hematoxylin and eosin staining was performed following Sur et al 2025 (38). Brightfield images were captured at 100× (10× eyepiece * 10× objective lens) and 200× (1 × eyepiece * 20× objective lens) magnification and analyzed using QuPath software. The renal corpuscle number (RCN) was counted from 5 images at 100× magnification and averaged to get the mean values for each hamster. The area (μm^2^, renal corpuscle area [RCA]) and perimeter (μm, RCP) of cortical renal corpuscles and the diameter (μm) of the convoluted tubules (CT) were measured from 10 images at 200× magnification and averaged to obtain the mean values.

### RNA Extraction and Quantitative PCR

Quantitative PCR (qPCR) assay was conducted on select pituitary gland samples obtained from experiment 1 (LP, SP12wk, SP28wk; N = 6) to confirm seasonal changes in *Prl* mRNA expression. RNA was extracted from the pituitary gland tissue using a QIAGEN RNeasy Plus Mini Kit (Manchester, UK) (38). Nanodrop (ND-1000, Thermoscientific) was used to estimate RNA concentration and 260/280 spectra absorbance values. QuantiTect Reverse Transcription Kit (Qiagen) was used to synthesize the cDNA from 1 μg of RNA and stored at −20 °C until qPCR. A 10-μL reaction/well consisted of 5 μL SYBR Green PCR master mix and primer mix and 5 μL of cDNA (100 ng). The qPCR was run in duplicate with negative controls for each gene on Stratagene MX3000. 18 seconds rRNA transcript was used as the reference gene following previous publications that confirmed the efficacy of the 18 seconds rRNA transcript in photomanipulation studies (39). qPCR procedure consisted of an initial denaturation at 95 °C for 10 minutes, succeeded by 40 cycles of denaturation at 95 °C for 30 seconds, primer-specific annealing temperature for 30 seconds, and an extension step at 72 °C for 30 seconds. The qPCR reaction was terminated at 95 °C for 1 minute. The reaction specificity was confirmed by the melt curve assay. The primer sequences and their annealing temperatures are listed in Table S1 (40). PCR Miner determined the efficiency and Ct values (41). The samples were assessed following Minimum Information for Quantitative Real-Time PCR (42). ΔΔCt method was used to determine the fold change values of the transcripts.

### Minion Transcriptome Sequencing

Transcriptome sequencing was conducted on kidney samples obtained from experiment 1 (LP, SP12wk, SP28wk; N = 4, each group) and experiment 2 (SAL, PRL; N = 6, each group). RNA was extracted using QIAGEN RNeasy Plus Mini Kit (Manchester, UK) (38, 43). Nanodrop (ND-1000, Thermoscientific) determined the RNA concentration and 260/280 values. The 260/280 absorbance values were ∼2.0 for all samples. Oxford Nanopore Direct cDNA Native Barcoding (SQK-DCS109 and EXP-NBD104) was used to synthesize cDNA following the manufacturer's protocol. One Spot-ON Flow cell (R9 version FLO-MIN106D) was used for each experiment. A single flow cell had N = 12 samples obtained from experiment 1 (N = 4, each group) and experiment 2 (N = 6, each group) for kidney tissue. Sequencing was performed using MinION Mk1B (MN26760, Oxford Nanopore Technologies). The sequencing assays were run for 72 hours at −180 mV voltage, and fast5 files were generated in single folders for downstream bioinformatic analyses.

### Bioinformatic Analyses

The steps for the transcriptomic analyses are provided in Supplementary Fig. S1 (40). The data analysis was performed in R-studio and run in the Conda package manager. The fast5 files were basecalled and demultiplexed using Guppy (4.2.1). The adapters were removed with Porechop (v0.2.4), and reads were selected for long-reads using Filtlong (v0.2.0; minimum 25 bases, mean q value = 9). Mus musculus reference genome (NCBI RefSeq assembly: GCF_000001635.27) was used to align the transcripts using Minimap2 (v2.17) (44). Weighted gene coexpression network analyses (WGCNA) were conducted in R-studio to identify coexpression modules, and a cluster dendrogram and a heatmap of module-trait relationships were plotted (38). The counts per million (CPM) values of genes from modules having a significant relationship (*P* ≤ .05) with CT diameter were plotted thereafter. ShinyGO (0.77) performed the functional annotation and pathway enrichment of the transcripts identified in the significant modules (Fig. S2) (40). Venny (2.1.0) was used to generate Venn diagrams identifying common genes linked with specific physiological indices between experiment 1 and experiment 2.

### General Statistical Analyses

Shapiro-Wilk test determined the normality of the data, following which either parametric or nonparametric tests were performed. Significance for all analyses was determined at *P* ≤ .05. The figures were generated using BioRender.com (38).

#### Statistical analyses for experiment 1

One-way ANOVA was performed on water intake, kidney mass, kidney volume, RCA, and *Prl* gene expression, followed by Tukey post hoc test. Kruskal-Wallis test was performed on RCP, RCN, and CT diameter, followed by Dunn post hoc test.

#### Statistical analyses for experiment 2

Unpaired Student *t*-test was performed on kidney mass, kidney volume, kidney somatic index, RCA, RCP, and RCN. Mann-Whitney test was performed on CT diameter.

## Results

Raw data are available in Table S2 (40). Sequencing data are available in the Gene Expression Omnibus database GSE292481. R-codes for bioinformatic analyses are available in Table S3 (40).

### Experiment 1: Seasonal Adaptations in Water Homeostasis and Renal Physiology

#### Seasonal adaptations in kidney mass and convoluted tubule diameter despite consistent water intake

**Figure 1.**
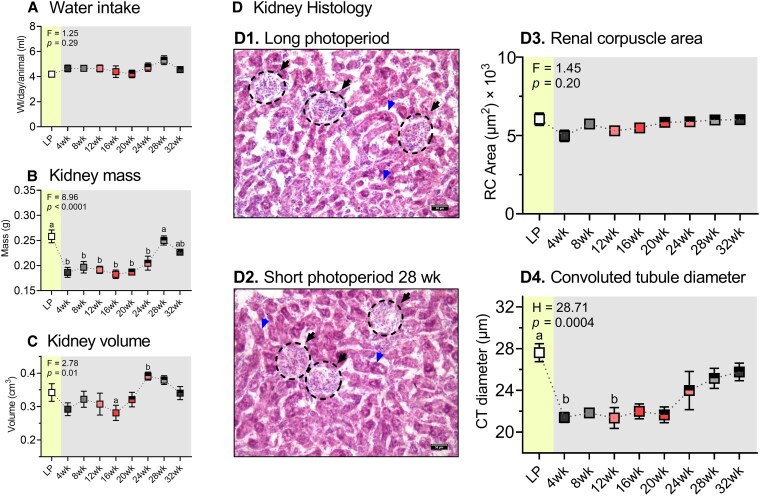
Photoinduced changes in drinking behavior, renal morphometry, and histology. Mean ± SEM (N = 6) values of the water intake (A), kidney mass (B), and kidney volume (C) across phototreatment groups (LP, long photoperiod; SP, short photoperiod, 4-32 weeks). Histological representations of the renal cortex in long photoperiod (D1) and short photoperiod 28 weeks (D2). The black and blue arrowheads denote renal corpuscle and convoluted tubules, respectively. Mean ± SEM (N = 6) values renal corpuscle area (D3) and convoluted tubule diameter (D4) across phototreatment groups. Letters represent significant differences in mean values. Statistical significance was determined at *P* ≤ .05.

Water intake showed no significant changes across the phototreatment groups (Fig. 1A; *P* > .05, 1-way ANOVA). One-way ANOVA revealed a significant effect of phototreatment on the kidney mass (Fig. 1B; F = 8.96, *P* < .0001) and kidney volume (Fig. 1C; F = 2.78, *P* = .01). Compared to LP, kidney mass decreased between SP 4 to 24 weeks and showed a spontaneous increase at SP 28-week exposure (*P* < .05, Tukey post hoc test). There was a significant difference in kidney volume between SP 16 and 24 weeks (*P* < .05, Tukey post hoc test). There were no significant changes in the renal corpuscle area (Fig. 1D3; *P* > .05, 1-way ANOVA), renal corpuscle perimeter, or corpuscle number (Fig. S3A-B; *P* > .05, Kruskal-Wallis test) (40). A Kruskal-Wallis test revealed a significant effect of phototreatment on the convoluted tubule diameter (Fig. 1D4; H = 28.71, *P* = .0004). Compared to LP, convoluted tubule diameter was reduced in SP at 4 and 12 weeks (*P* < .05, Dunn post hoc test).

#### Seasonal changes in renal molecular architecture

**Figure 2.**
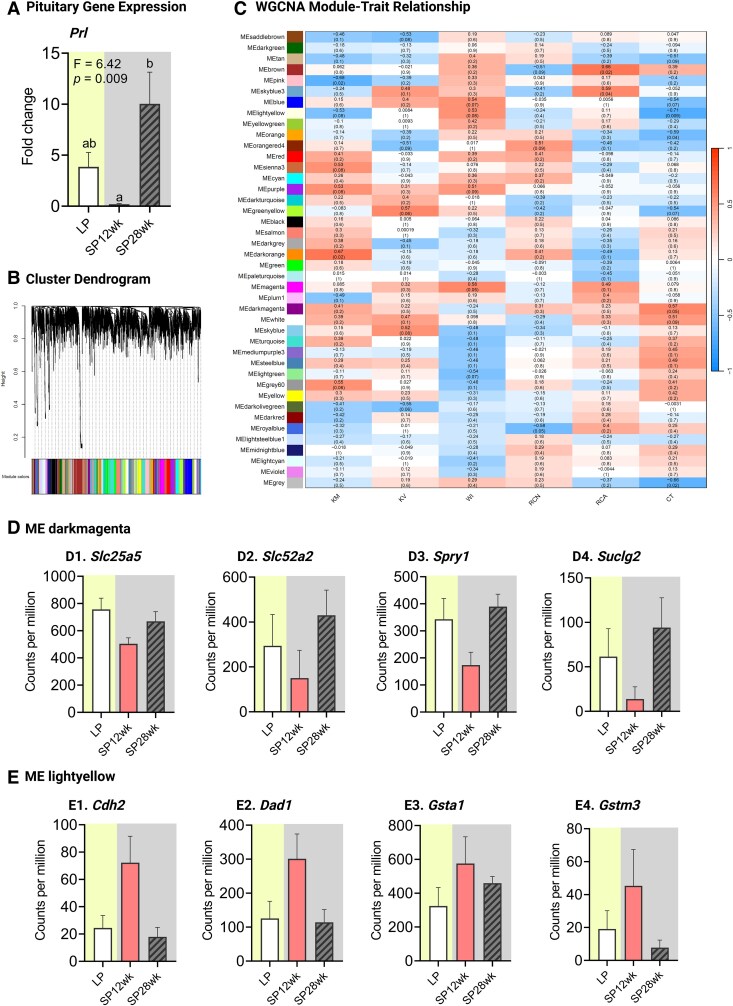
Photoinduced changes in hypophyseal and renal transcript levels. (A) Bar plot depicting transcript expression (mean ± SEM, N = 6) of *Prl* (*P* = 0.009, 1-way ANOVA) across phototreatment groups (LP, long photoperiod; SP, short photoperiod, 12-28 weeks) by qPCR assay (B) Cluster dendrogram representing coexpression modules derived from weighted gene coexpression network analysis (WGCNA) of kidney transcriptomic data from experiment 1. Each module is color-coded to indicate distinct gene clusters. (C) WGCNA module-trait relationships display the correlations between gene modules and physiological traits. (D-E). Bar plots depicting mean ± SEM (N = 4) of the counts per million (CPM) values of *Slc25a5*, *Slc52a2*, *Spry1*, and *Suclg2* gene from module darkmagenta (D1-D4) and *Cdh2*, *Dad1*, *Gsta1,* and *Gstm3* gene from module lightyellow (E1-E4) across phototreatment groups. Abbreviations: CT, convoluted tubule width; KM, kidney mass; KV, kidney volume; RCA, renal corpuscle area; RCN, renal corpuscle number; WI, water intake.

WGCNA identified 43 coexpression modules, of which 10 module-trait relations were significant, including 5 positive and 5 negative correlations (Fig. 2B–2C). Module-CT relationship revealed a strong relationship between Module darkmagenta and CT diameter, sharing a common branch (Fig. S2A1) (40). Module darkmagenta contained 57 transcripts and was positively associated with CT diameter (Table S4; R^2^ = 0.57, *P* = .05) (40). ShinyGO analyses of the transcripts of the Module darkmagenta identified enrichment of mitochondrial ADP transport, tricarboxylic acid cycle, mitochondrial electron transport, and endocytosis pathways (Fig. S2A2a) (40). Specifically, the expression of Solute Carrier Family 25 Member 5 (*Slc25a5*), Solute Carrier Family 52 Member 2 (*Slc52a2*), Sprouty RTK Signaling Antagonist 1 (*Spry1*), and Succinate-CoA Ligase GDP-Forming Beta Subunit (*Suclg2*) were correlated with CT diameter (Fig. 2D1-4). Module lightyellow contained 79 transcripts and was negatively associated with CT diameter (Table S4; *R*^2^ = −0.71, *P* = .009) (40). ShinyGO analyses of the transcripts of the Module lightyellow identified enrichment of peptide metabolic processes, negative regulation of cell death, and proton transmembrane transport (Fig. S2A2b) (40). The expression of Cadherin 2 (*Cdh2*), Defender Against Cell Death 1 (*Dad1*), Glutathione S-Transferase Alpha 1 (*Gsta1*), and Glutathione S-Transferase Mu 3 (*Gstm3*) were correlated with CT diameter (Fig. 2E1-4).

#### Prolactin mRNA expression varies across photoinduced seasonal rhythm

One-way ANOVA revealed a significant effect of phototreatment on *Prl* expression (Fig. 2A; F = 6.42, *P* = .009). *Prl* expression was similar between LP and SP 28 weeks and showed downregulation in the SP 12 weeks (*P* < .05, Tukey post hoc test).

### Experiment 2: Prolactin Induces Seasonal Changes in the Kidney

#### Prolactin administration increased kidney mass and convoluted tubule diameter

**Figure 3.**
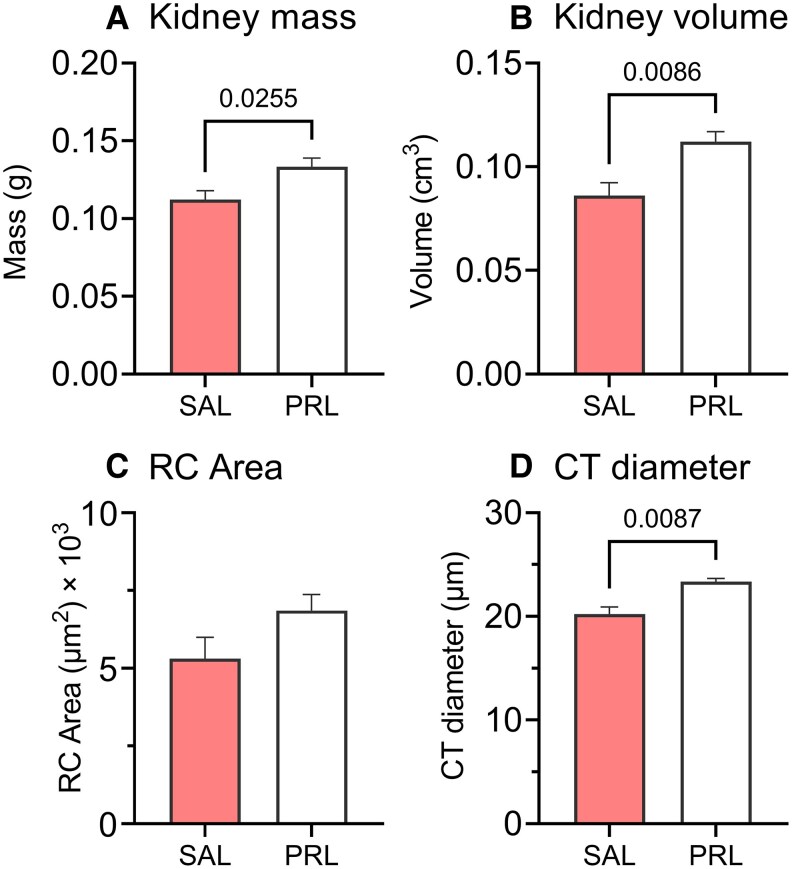
Prolactin-induced changes in renal morphometry and histology. Mean ± SEM (N = 6) values of the kidney mass (A), kidney volume (B), renal corpuscle area (C), and convoluted tubule diameter (D) across saline (SAL) and prolactin (PRL) treatment groups. The number above the bar represents a significant *P* value. Statistical significance was determined at *P* ≤ .05.

Student *t*-test revealed a significant increase in kidney mass (Fig. 3A; t = 2.62, *P* = .02) and kidney volume (Fig. 3B; t = 3.25, *P* = .008) in response to intraperitoneal injections of prolactin, compared to the control group. There were no significant differences in kidney somatic index, RCA, RCP, and RCN between the SAL and PRL groups (Fig. 3C, Fig. S3C-E; *P* > .05, unpaired Student *t*-test) (40). Mann-Whitney (MW-U) test revealed a significant increase in convoluted tubule diameter in PRL, compared to the SAL group (Fig. 3D; MW-U = 2, *P* = .008).

#### Regulation of the renal molecular architecture

**Figure 4.**
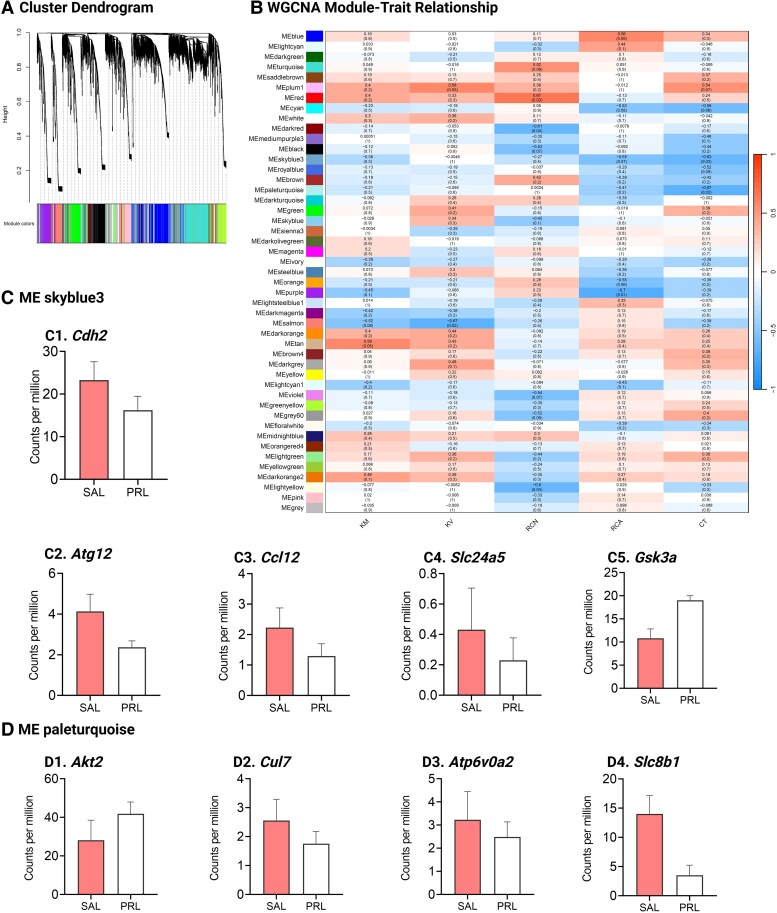
Prolactin-induced changes in renal transcript levels. (A) Cluster dendrogram representing coexpression modules derived from weighted gene coexpression network analysis (WGCNA) of kidney transcriptomic data from experiment 2. Each module is color-coded to indicate distinct gene clusters. (B) WGCNA module-trait relationships display the correlations between gene modules and physiological traits. (C-D). Bar plots depicting mean ± SEM (N = 6) of the counts per million (CPM) values of *Cdh2*, *Atg12*, *Ccl12*, *Slc24a5*, and *Gsk3a* gene from module skyblue3 (C1-C5) and *Akt2*, *Cul7*, *Atp6v0a2* and *Slc8b1* gene from module pale turquoise (D1-D4). Abbreviations: CT, convoluted tubule width; KM, kidney mass; KV, kidney volume; RCA, renal corpuscle area; RCN, renal corpuscle number.

WGCNA identified 48 unique coexpression modules, where 9 module-trait relations were significant, including 3 positive and 6 negative correlations (Fig. 4A–4B). Module-CT relationship did not reveal a strong positive relationship between modules and CT diameter (Fig. S2B1) (40). Module skyblue3 contained 96 transcripts and was negatively associated with CT diameter (Table S5; *R*^2^ = −0.63, *P* = .03) (40). ShinyGO analyses of the transcripts of the Module skyblue3 identified enrichment of cytoskeletal organization, protein transport, cell migration, and protein ubiquitination pathways (Fig. S2B2a) (40). Specifically, the expression of Cadherin 2 (*Cdh2*), Autophagy Related 12 (*Atg12*), C-C Motif Chemokine Ligand 12 (*Ccl12*), Solute Carrier Family 24 Member 5 (*Slc24a5*), and Glycogen Synthase Kinase 3 Alpha (*Gsk3a*) were negatively correlated with CT diameter (Fig. 4C1-5). Module paleturquoise contained 113 transcripts and was negatively associated with CT diameter (Table S5; *R*^2^ = −0.67, *P* = .02) (40). Specifically, the expression of AKT Serine/Threonine Kinase 2 (*Akt2*), Cullin 7 (*Cul7*), ATPase H + Transporting V0 Subunit A2 (*Atp6v0a2*), and Solute Carrier Family 8 Member B1 (*Slc8b1*) were correlated with CT diameter (Fig. 4D1-4).

### Identification of Common Genetic Regulators of Renal Physiology

**Figure 5.**
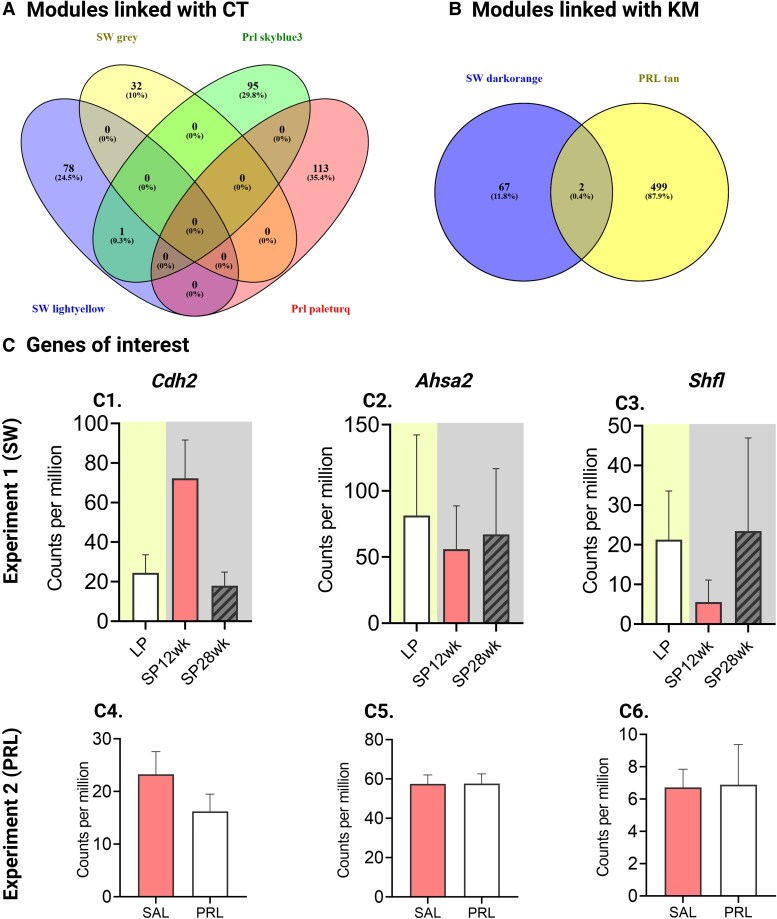
Shared molecular drivers of kidney function. (A-B). Venn diagrams illustrating overlapping genes associated with convoluted tubule width (CT) and kidney mass (KM) across experiments 1 and 2. In experiment 1, CT-associated modules include SW light yellow and SW gray, and KM-associated module includes SW dark orange. In experiment 2, CT-associated modules are Prl skyblue3 and Prl pale turquoise, while KM-associated module is Prl tan. (C) Bar plots depicting mean ± SEM of the counts per million (CPM) values of *Cdh2*, *Ahsa2*, and *Shfl* gene in experiment 1 (LP, long photoperiod; SP, short photoperiod, SP12-28 weeks; N = 4) and experiment 2 (PRL, prolactin; SAL, saline; N = 6).

Venny (2.1.0) compared the transcripts of grey and lightyellow modules (experiment 1) with skyblue3 and paleturquoise modules (experiment 2) (Fig. 5A) and identified Cadherin 2 (*Cdh2*) (Fig. 5C1, 5C4) as a common gene negatively linked with convoluted tubule diameter. Comparison between module darkorange (experiment 1) and module tan (experiment 2) (Fig. 5B) identified Activator of HSP90 ATPase Homolog 2 (*Ahsa2*) (Fig. 5C2, 5C5) and Shiftless Antiviral Inhibitor of Ribosomal Frameshifting (*Shfl*) (Fig. 5C3, 5C6) as common genes positively linked with kidney mass. No common genes were identified to be linked with the renal corpuscle number between module royalblue (experiment 1) and module darkred and lightyellow (experiment 2) (Fig. S2C) (40).

## Discussion

The present study demonstrates robust photoperiod adaptations in renal morphology and transcriptome architecture in Djungarian hamsters. Long photoperiods induced renal hypertrophy, whereas short photoperiods induced a transient reduction, followed by an endogenous recovery in kidney mass and CT diameter. Transcriptomic analyses identified gene modules correlated with CT diameter enriched in pathways related to mitochondrial function, solute transport, and cellular turnover. The long photoperiod-induced upregulation of *Slc25a5*, *Suclg2*, and *Spry1* genes indicates enhanced renal metabolic activity and solute handling, while negative associations with *Cdh2* and *Gstm3* transcripts reveal active remodeling and protective antioxidant responses under short photoperiods. PRL injections in the short photoperiod-housed hamsters induced renal hypertrophy simulating long photoperiodic induction. Modules negatively correlated with CT diameter were enriched for genes involved in cytoskeletal organization, protein transport, and cellular remodeling, including *Cdh2*, *Atg12*, *Ccl12*, *Slc24a5*, *Gsk3a*, and *Akt2*, revealing the transcriptional basis for PRL-mediated structural modulation. Overall, these results suggest that renal plasticity is governed by photoresponsive transcriptional programs that coordinate seasonal changes in kidney structure and function.

### Photoinduced Transcriptional Modulation Underlies Seasonal Renal Plasticity

Photoperiodic treatment in hamsters induced a distinct seasonal rhythm in kidney mass and volume. Similar annual cycles in kidney mass have been observed in caribou (*Rangifer tarandus*) (45), red deer (*Cervus elaphus*) (46), sheep (*Ovis aries*) (47), and Indian mongoose (*Herpestes auropunctatus*) (48). The larger renal dimensions under long photoperiods likely reflect seasonal adaptations that support increased metabolic and reproductive demands. In mongoose, the lack of water availability during the dry season is proposed to induce kidney involution, whereas seasonal reproductive activity was associated with increased kidney mass (48). The short photoperiod-induced acute reduction and the programmed recovery of kidney mass are driven by an endogenous timer in anticipation of seasonal transitions (25). The absence of seasonal change in water intake challenges the functional advantage of seasonal adaptations in the kidney function. Reductions in organ size likely facilitate energy conservation during periods of low physiological demand. This aligns with the complex energy-saving strategies employed by Djungarian hamsters to endure harsh winter conditions (16). During the reproductive season, hamsters survive with limited rainfall and produce highly concentrated urine, indicating seasonal variations in renal function (49, 50). Future studies assessing whole-body water content and urinary output will aid in the understanding of the significance of seasonal kidney changes.

Histomorphometric analysis revealed seasonal changes in convoluted tubule diameter, mirroring the kidney mass change across phototreatments. In rodents, convoluted tubules occupy much of the renal cortex, with the proximal segment comprising 48% to 76% (51, 52), and the distal about 3% to 5% of cortical volume (53, 54). The proximal tubule reabsorbs the majority of water and solutes and secretes organic compounds, while the distal tubule fine-tunes electrolyte balance through selective reabsorption and secretion (52). In the current study, the expression of *Slc25a5*, *Suclg2*, *Slc52a2*, and *Spry1* genes was positively correlated with the convoluted tubule width. SLC25A5, a mitochondrial ADP/ATP translocase, facilitates ATP exchange across the mitochondrial inner membrane for active tubular reabsorption (55). SUCLG2 encodes subunit β of succinyl-CoA synthetase, a key enzyme in the tricarboxylic acid cycle (56), whereas SLC52A2 transports riboflavin, a key cofactor in oxidative metabolism (57, 58). The kidney relies on a high mitochondrial abundance to fuel waste removal and maintain fluid and electrolyte balance (59). Therefore, during short photoperiods, reductions in tubular dimensions are likely accompanied by attenuated mitochondrial and transport activity, reflecting the decreased metabolic demand. Structural remodeling of the kidney parallels these functional changes, as indicated by the downregulation of *Spry1*, a negative regulator of receptor tyrosine kinase signaling, including the FGF and GDNF pathways essential for kidney development (60, 61).

*Dad1*, *Gsta1*, and *Gstm3* expressions were negatively correlated with the CT diameter change. DAD1, a subunit of the oligosaccharyltransferase complex, functions as a negative regulator of programmed cell death linked to endoplasmic reticulum stress-induced apoptotic pathways (62). GSTA1 and GSTM3, both glutathione S-transferases, are key to cellular detoxification and oxidative stress responses (63). Studies in small mammals suggest that prolonged light exposure elevates renal oxidative stress, whereas extended periods of darkness contribute to reduced oxidative damage (64, 65). Likewise, melatonin treatment reduced malondialdehyde, a marker of cellular peroxidative in the renal tissues of rabbits (*Oryctolagus cuniculus*) (66). Exposure to short photoperiods in the striped dwarf hamster (*Cricetulus barabensis*) decreased oxidative stress in hamster kidneys through the Nrf2-Keap1 signaling pathway (67). The short photoperiod-induced upregulation of stress-responsive genes in the current study likely enhances antioxidant defenses and protects against cellular stress in hamsters.

### Prolactin Mediates Seasonal Remodeling of Renal Structure and Gene Expression

Hypophyseal *Prl* expression showed an acute decrease (12 weeks), followed by a sharp endogenous increase on prolonged exposure (28 weeks) to short photoperiods (Fig. 2A). This aligns with previous findings across a wide range of eutherian and metatherian mammals in which elevated summer PRL levels regulate seasonal changes in metabolism, reproduction, and parental behavior (68). Notably, although renal *Prl* expression is reported in mice (31), it is not expressed in the Djungarian hamster kidneys (27), suggesting no direct action of renal PRL in the photoperiodic response. PRL injection in short photoperiod-housed hamsters induced renal hypertrophy and increased convoluted tubule diameter, corroborating photoinduced seasonal changes in kidney morphology observed in experiment 1. Likewise, in female rats during lactation, a hyperprolactinemic state, the kidneys undergo enhanced growth, including elevated glomerular filtration rate as well as increased salt and water reabsorption (69). Genes from module skyblue3 *(Atg12, Ccl12, Slc24a5)* and module paleturquoise *(Cul7, Atp6v0a2, Slc8b1*) were downregulated during tubular hypertrophy. ATG12 forms a conjugated complex with ATG5/ATG16L1 essential for autophagosome formation and regulates autophagy which is critical for kidney homeostasis (70). ATP6V0A2, a subunit of V-ATPase, is also essential for autophagy and regulates lysosomal and endosomal acidification to degrade cellular components (71). In nutrient-deficient environments, mammalian autophagy promotes renal ketogenesis for energy (72). Short photoperiod exposure in striped dwarf hamsters also induced autophagolysosome formation in photo-responsive organs such as the Harderian gland (73). Further, PRL injections in the present study downregulated *Cul7* expression, which encodes cullin-7, a key component of the ubiquitin-proteasome system (74). PRL thus promotes anabolic pathways that support renal growth and tubular enlargement, while suppressing organ-specific catabolic processes like autophagy and protein degradation crucial in winter-like conditions. PRL also maintains a low inflammatory state, as indicated by the downregulation of *Ccl12*, a chemoattractant that mediates inflammatory responses by promoting the migration of leukocytes (75). Both the solute carrier (SLC) ion transporters identified function as sodium-calcium exchangers, where SLC24A5 regulates pigmentation (76), wheresa SLC8B1 is responsible for mitochondrial Ca²⁺ extrusion in excitable cells (77). Downregulation of these genes during tubular growth suggests a reduction in nonessential calcium transport activity, aiding the regulation of Ca²⁺ homeostasis. As only females were used in experiment 2, it remains possible that other transcripts associated with the photoperiodic regulation of water rheostasis in male hamsters are underrepresented. Furthermore, bromocriptine-mediated PRL suppression in LP-housed Djungarian hamsters did not alter the kidney mass (8). These data suggest that either prior exposure to long photoperiod induces a long-term programmed effects on renal function or that PRL is not necessary to maintain higher kidney mass. Other hormonal pathways such as GH signaling may facilitate the photoperiod regulation of kidney function.

### Cadherin 2: A Candidate for Prolactin-driven Seasonal Tubule Remodeling

Renal *Cdh2* expression was suppressed by both long photoperiod exposure and PRL treatment, and it exhibited a negative correlation with tubular diameter. Cadherins are a family of calcium-dependent transmembrane proteins essential for cell adhesion and signaling, and they play a critical role in establishing renal epithelial polarity through homophilic interactions between the extracellular domains of adjacent cells (78). *Cdh2* encodes N-cadherin protein that is predominantly expressed in the proximal tubule (79). N-cadherin is markedly reduced in proximal tubules during acute kidney injury, suggesting a role in epithelial integrity (80). In long photoperiods, reduced *Cdh2* expression may alter cell-cell adhesion, resulting in tubule dilation and increased width. During winter hibernation in the dormouse (*Muscardinus avellanarius*), proximal convoluted tubule cells retain intact ultrastructure and polarity, along with a hypertrophied apical endocytic apparatus (81). Similar changes in tubular cells have been reported in garden dormice (*Eliomys quercinus*) (82) and golden-mantled ground squirrels (*Callospermophilus lateralis*) (83) during hibernation or torpor. Hibernators potentially upregulate protective factors that preserve cellular homeostasis and structural integrity during torpor and arousal (84). In response to winter-like conditions, CDH2 likely contributes to epithelial cohesion and preserves cellular architecture in torpid Djungarian hamsters. *Ahsa2* and *Shfl* expressions were positively correlated with kidney mass in both experiments, indicating their potential roles in renal growth and remodeling. AHSA2 is a member of the AHA family and functions as a cochaperone that enhances the ATPase activity of Hsp90, a key heat shock protein involved in protein folding and stress responses (85). SHFL is known to inhibit programmed-1 ribosomal frameshifting, a mechanism that viruses exploit to regulate their protein synthesis (86). Beyond its antiviral function, SHFL may regulate host protein translation, impacting cellular proliferation and growth in the kidney.

## Conclusion

The present study demonstrates that photoperiodic cues drive seasonal remodeling of kidney structure and transcript levels in Djungarian hamsters. Long photoperiods induced renal hypertrophy and tubular expansion which corresponded to increased mitochondrial and transport activity. Short photoperiods triggered a transient reduction followed by endogenous recovery in renal dimensions, highlighting an internal circannual timer regulating the renal growth-regression cycle. Markers for antioxidant defense and protection against cellular stress were upregulated in the winter short photoperiod. PRL signals the seasonal transitions to the kidney to induce renal growth and attenuates anabolic pathways including autophagy. *Cdh2*, encoding N-cadherin, emerged as a key regulator. Its downregulation during long photoperiods and PRL-induced hypertrophy, along with its inverse relationship with tubular dimensions, suggests that reduced CDH2 may promote epithelial remodeling and structural expansion, while in winters it aids epithelial cohesion. This pattern mirrors seasonal adaptations in hibernators, where epithelial integrity is preserved throughout torpor. These findings offer new insight into programmed renal rheostasis and the molecular underpinnings of seasonal renal plasticity.

## Data Accessibility

Original data generated and analyzed during this study are included in this published article or available online (40).

## Abbreviations

CPM: counts per million
CT: convoluted tubule
LP: long photoperiod
PRL: prolactin
qPCR: quantitative PCR
RCA: renal corpuscle area
RCN: renal corpuscle number
RCP: renal corpuscle perimeter
SP: short photoperiod
WGCNA: weighted gene coexpression network analyses
